# Lipid Profiles and Prevalence of Dyslipidemia in Eastern Iranian Adolescents, Birjand, 2012

**Published:** 2015-07

**Authors:** Fatemeh Taheri, Tayebeh Chahkandi, Toba Kazemi, Bita Bijari, Mahmoud Zardast, Kokab Namakin

**Affiliations:** Birjand Atherosclerosis and Coronary Artery Research Center, Birjand University of Medical Sciences, Birjand, Iran

**Keywords:** Dyslipidemia, Adolescents, Iran

## Abstract

**Background:**

Cardiovascular risk factors begin in childhood and adolescence. This study aimed at assessing serum lipids and prevalence of Dyslipidemia in 11-18 year old students of Birjand.

**Methods:**

The present cross-sectional, descriptive, and analytical study was done on 2,643 middle and high school students of Birjand aged 11-18 years (1,396 girls and 1,247 boys). Blood samples were collected for the measurement of blood lipids, including Cholesterol, Triglyceride, HDL, and LDL after a 12-hour fasting period. The defined borderline and abnormal values stated in 2011 by the American Academy of Child, was used.

**Results:**

According to our results, it is concluded that: (i) 34.3% (31.3% girls and 37.6% boys) of adolescents had at least one dyslipidemia. (ii) 24.7% of the individuals had HDL lower than 40, where 14% of them TG≥130, 6.1% of cases TC≥200, and 3.5% of cases LDL≥130. Lipid disorder within low HDL type and hypertriglyceridemia were significantly higher in boys (P<0.05) than girls. Hypercholesterolemia and hypertriglyceridemia in the age group of 11-14 years and low HDL in the age group of 15-18 years showed the highest values (P<0.05).

**Conclusion:**

Adolescents of Birjand have high prevalence of dyslipidemia. Preventive measures are recommended to improve lifestyle, including healthy nutrition, encouraging adolescents to exercise, and more mobility.

## Introduction

The major cause of adult death in the world is cardiovascular disease (CVD) and is increasing in many of the developing countries such as Iran.^1-3^ Dyslipidemia or abnormal levels of blood lipids, such as hypercholesterolemia, begins from childhood and adolescence and can lead to premature atherosclerosis.^4-6^ There are some evidences of atherosclerosis in children. Bugalus Heart Study Autopsy (BHSA) researches showed that high total cholesterol (TC), low-density lipoprotein (LDL), and low levels of high-density lipoprotein (HDL) are associated with the increased coronary artery lesions.^7-9^ Half of the children with dyslipidemia will suffer from dyslipidemia in adulthood. Moreover, atherosclerotic plaques increase with age.^4^^,^^7^ It is known that dyslipidemia in children and adolescents act as in adults, which is related with other cardiovascular risk factors such as hypertension and obesity.^10^^,^^11^ Therefore, lipid screening for the detection and control of dyslipidemia in children and adolescents and CVD risk reducing, is recommended. The National Heart, Lung, and Blood Institute (NHLBI) offer guidelines for lipid screening in children and adolescents.^4^ Serum lipids levels are related to sex, race, and the age of children and adolescents.^4^ Due to reduced consumption of traditional foods as well as urbanization, industrialization, and increased fatty foods and fast-food, the average cholesterol levels of children and adolescents of many countries is increasing.^12-14^ In Iran, like many other developing countries, the increase of CVD is a public health issue.^15^

Similar to other countries, cardiovascular risk factors including obesity are growing among the Iranian children and adolescents. Dyslipidemia and other risk factors are increasing parallel with the increase of obesity.^16^ Previous studies have reported high triglycerides and low HDL among the Iranian adolescents in comparison with the Americans and other nations.^16^ The correction and control of serum lipids of children and adolescents is possible and can reduce long-term complications in adulthood.^4-6^ In order to assess and understand the current situation with dyslipidemia prevalence, serum lipids evaluation in children and adolescents of different parts of Iran is essential. Then, based on the obtained data, planning and intervention measurements will help reduce cardiovascular mortality in adulthood. Therefore, the study of lipid profile and the prevalence of dyslipidemia especially in children and adolescents are important. This study aimed to determine the prevalence of dyslipidemia and conduct lipid profile in 11-18 years old adolescents of Birjand.

## Subjects and Methods

The present cross-sectional, descriptive and analytical study was done during the year 2012 on 2,643 middle and high school students of Birjand aged 11-18 years (1,396 girls and 1,247 boys). The sample size was determined based on the formula from Fesharakinia study:^17^

$$n=\frac{Z_{1-\alpha /2}.P(1-P)}{d^{2}}$$

where α=0.05, Z_1-α/2_=1.96, P=0.04 (prevalence dyslipidemia=4%), d=0.01, and n=752 (for comparison between two sexes, n=752×2=1,504).

We considered 1,500 individuals for each education level (middle and high schools). The samples were selected through multistage sampling. Considering the distribution of schools in different districts of the city, at first 14 girls schools and 14 boys schools, each consisting 7 middle and 7 high schools, were selected. Then, with respect to the population of each school and its ratio to the total number of students in that class, some students from each class were selected. At first, 3,000 students (1,500 in middle and 1,500 in high schools) were chosen and a demographic questionnaire together with a consent form was sent to their parents. The parents were requested to fill out the demographic and consent forms and return them to the school if they agree with their child’s participation in the plan. The exclusion criteria were, not having any chronic disease or endocrine disorder such as diabetes and not being on a treatment of corticosteroids or drugs influencing lipid profile. In the next step, after getting the permission from the education office and appropriate coordination, two trained nurses visited the schools to collect and record the demographic information. Eventually, 2,643 individuals, after the exclusion of a few with incomplete information, were selected for the analysis. To measure blood lipids, including cholesterol, triglyceride, HDL, and LDL, blood samples were derived from the cubital vein of the left hand after a 12 hour fasting. The blood sample was taken in 5 ml vacuum tubes containing separator gel and clot activator manufactured by Bacton Dickinson (UK). The obtained samples were immediately centrifuged and their respective lipid levels were determined by applying enzymatic procedure using German Rosh kits, with Biochemical Autoanalyser Prestige 24i (Japan).

*Definition of Pediatric Dyslipidemia*

NHLBI panel definition of dyslipidemia in 2011 was used in our study.^4^ These cutoff points delineate lipid values as borderline and abnormal were:

1. Borderline: HDL<45 mg/dl, LDL≥100 mg/dl, TC≥170 mg/dl and TG>130 mg/dl
2. Abnormal: HDL<35 mg/dl, LDL≥130 mg/dl, TC≥200 mg/dl TG>130 mg/dl

The data were analyzed using the SPSS software, version 15. Analysis between categorical variables was assessed by the Chi-square test. The independent *t* test was used to compare the means between the two groups. While α≤0.05 was taken as the significant level.

## Results

In the present study, 2,643 students in the age group of 11-18 with an average 14.5±2 years, including 1,396 (52.5%) girls and 1,247 (47.2%) boys were included (table 1). Most of the students aged 14 (18.7%) and 12-13 years (17.7%). The mean weight, stature, waist circumference, and BMI were measured 49.78±13.22 kg, 156.20±9.68 cm, 68.43±9.75 cm, and 20.21±4.02, respectively. The minimum and maximum of these quantities were determined as 27 and 117 kg, 130 and 187 cm, 47 and 114 cm, and 12.5 and 40.44, respectively. In 904 (34.2%) adolescents, there was at least one lipid disorder (abnormal or borderline) with the order of prevalence in individuals as 42.6% for HDL<45 mg/dl, 39.6% for TG≥100 mg/dl, 24% for TC≥170 mg/dl, and 11.2% for LDL≥100 mg/dl. Another order of lipid prevalence was 24.7% for HDL<40 mg/dl, 14% for TG≥130 mg/dl, 6.1% for TC≥200 mg/dl, and 3.5% for LDL≥130 mg/dl. Table 2 represents the mean of serum lipids with respect to sex. The mean age of boy and girl students were 14.4±1.9 and 14.5±2.1, respectively, without significant difference (t=-0.18, P=0.06). The stratified analysis was used to eliminate the confounding effect of obesity. Students having normal BMI and those suffering from overweight and obesity were analyzed separately for comparison of the mean serum lipids and dyslipidemia in both sexes. Girl students with normal weight had significantly higher levels of mean TC and LDL compared with boys. Mean TG was higher in boys compared with girls; but this difference was not statistically significant. Mean HDL was higher in girls compared with boys; but the difference between the two was not statistically significant. Mean serum lipids of students suffering overweight and obesity did not show significant statistical difference in both genders (table 2). Table 3 shows the prevalence of abnormal values of TC and borderline for LDL, HDL, and TG with respect to sex. Boy students with normal weight had higher levels of Low HDL and hypertriglyceridemia compared with girls. The level of some blood lipids in overweight and obese students did not show a significant statistical difference in both sexes (table 3). Mean serum lipids in 11-14 and 15-18 years old were determined and then abnormal lipid disorders in both age groups were compared. It is noted that the mean of TC, LDL, HDL, and TG were significantly higher in 11-14 compared with 15-18 years old (table 4). Hypercholesterolemia and Hypertriglyceridemia were significantly higher in the age group of 11-14 years in comparison with 15-18, while low HDL was significantly higher in 15-18 than 11-14 age groups (table 5).

**Table 1:** Characterization of students

**Characteristics**		**Frequency**	**Percent**
Age group	11, 12y	705	26.7
	13, 14y	902	34.1
	15, 16y	687	26
	17, 18y	349	13.2
Sex	Male	1,247	47.2
	Female	1,396	52.8
BMI	Normal	2,231	84.4
	Overweight	198	7.5
	Obesity	214	8.1

**Table 2:** Mean of serum lipids according to sex in our cases

		**All (n=2,643)**	**Male (n=1,247)**	**Female (n=1,396)**	***t*** ** test**	**P value**
		**Mean±SD**	**Mean±SD**	**Mean±SD**		
Normal BMI	Total chol	152.58±28.67	150.52±29.88	154.42±27.44	-3.2	0.001
	LDL-C	83.63±22.63	82.51±22.35	84.63±21.93	-2.2	0.027
	HDL-C	46.98±10.47	46.62±10.95	47.30±10.01	-1.5	0.123
	Non-HDL-C	105.55±27.78	103.89±28.97	107.03±26.61	-2.6	0.008
	TG	93.32±45.95	95.29±51.31	91.57±40.53	1.8	0.06
Overweight and obese	Total chol	149.66±29.67	147.60±30.85	151.89±28.26	-1.4	0.16
	LDL-C	80.28±23.71	79.20±25.66	81.44±21.43	-0.91	0.36
	HDL-C	49.04±11.53	48.45±12.53	49.67±10.35	-1.02	0.30
	Non-HDL-C	100.62±27.80	99.14±2915	102.22±26.25	-1.07	0.28
	TG	84.24±37.71	84.44±42.42	84.02±32.00	0.10	0.91

Data compared by independent *t *test

**Table 3:** Prevalence of normal, borderline, and abnormal lipid levels according to sex in our cases

	**Criteria**		**All**	**Male**	**Female**	** X^ 2 ^**	**P value**
Normal BMI	TC	Acceptable	1,693 (75.9)	811 (77.2)	882 (74.7)	2.93	0.23
		Borderline	400 (17.8)	173 (16.4)	227 (19.3)		
		Abnormal	138 (6.2)	67 (6.4)	71 (6)		
	LDL-C	Acceptable	1,984 (88.9)	947 (90.1)	1,037 (87.9)	3.45	0.17
		Borderline	166 (7.5)	67 (6.4)	99 (8.4)		
		Abnormal	81 (3.6)	37 (3.5)	44 (3.7)		
	HDL-C	Acceptable	1,256 (56.3)	579 (55.1)	677 (57.3)	10.53	0.005
		Borderline	406 (18.2)	173 (16.5)	233 (19.8)		
		Abnormal	569 (25.5)	299 (28.4)	270 (22.9)		
	Non-HDL-C	Acceptable	1,652 (74)	795 (75.6)	857 (72.6)	2.69	0.25
		Borderline	398 (17.9)	175 (16.7)	223 (18.9)		
		Abnormal	181 (8.1)	81 (7.7)	100 (8.5)		
	TG	Acceptable	1,319 (59.1)	615 (58.5)	704 (59.6)	11.48	0.003
		Borderline	584 (26.2)	255 (24.3)	329 (27.9)		
		Abnormal	328 (14.7)	181 (17.2)	147 (12.5)		
Overweight and obese	TC	Acceptable	317 (76.9)	162 (82.6)	155 (71.8)	0.95	0.23
		Borderline	73 (17.8)	27 (13.8)	46 (21.3)		
		Abnormal	22 (5.3)	7 (3.6)	15 (6.9)		
	LDL-C	Acceptable	362 (87.8)	178 (90.7)	184 (85.1)	0.38	0.82
		Borderline	38 (9.3)	14 (7.2)	24 (11.1)		
		Abnormal	12 (2.9)	4 (2.1)	8 (3.8)		
	HDL-C	Acceptable	263 (63.8)	120 (61.2)	143 (66.3)	4.53	0.10
		Borderline	66 (16.1)	30 (15.3)	36 (16.6)		
		Abnormal	83 (20.1)	46 (23.5)	37 (17.1)		
	Non-HDL-C	Acceptable	308 (74.7)	158 (80.6)	150 (69.4)	0.73	0.69
		Borderline	76 (18.4)	29 (14.8)	47 (21.7)		
		Abnormal	28 (6.7)	9 (4.6)	19 (9)		
	TG	Acceptable	276 (66.9)	137 (69.9)	139 (64.3)	1.01	0.59
		Borderline	93 (22.6)	39 (19.9)	54 (25)		
		Abnormal	43 (10.5)	20 (10.2)	23 (10.7)		

Data compared by chi-square test

**Table 4:** Comparison of the mean serum lipids in two age groups (11-14 and 15-18 years)

	**All (n=2,643)**	**11-14 years (n=1,607)**	**15-18 years (n=1,036)**	***t*** ** test**	**P value**
	**Mean±SD**	**Mean±SD**	**Mean±SD**		
Total chol	152.58±29.11	154.95±28.26	148.89±30.02	-5.2	0.001
LDL-C	83.49±23.08	84.83±22.55	81.41±23.75	-3.7	0.001
HDL-C	47.29±10.65	48.12±10.92	46.00±10.10	-5.1	0.001
Non-HDL-C	105.24±2813	106.82±27.16	102.79±29.40	-3.6	0.001
TRIG	92.16±44.93	94.75±43.05	88.15±47.45	-3.6	0.001

Data compared by independent *t* test

**Table 5:** Prevalence of normal, borderline, and abnormal lipid levels in two age groups

**Criteria**		**All**	**11-14 years**	**15-18 years**	** X^ 2 ^**	**P value**
TC	Acceptable	2,010 (76)	1,181 (73.5)	829 (80)	14.75	0.001
	Borderline	473 (17.9)	319 (19.9)	154 (14.9)		
	Abnormal	160 (6.1)	107 (6.7)	53 (5.1)		
LDL-C	Acceptable	2,345 (88.7)	1,420 (88.3)	925 (89.4)	1.13	0.56
	Borderline	205 (7.7)	127 (7.9)	78 (7.5)		
	Abnormal	93 (3.5)	60 (3.8)	33 (3.1)		
HDL-C	Acceptable	1,519 (57.5)	969 (60.3)	550 (53)	21.03	0.001
	Borderline	472 (17.9)	290 (18)	182 (17.6)		
	Abnormal	652 (24.7)	348 (21.7)	304 (29.4)		
Non-HDL-C	Acceptable	1,960 (74.1)	1,165 (72.5)	795 (76.7)	5.89	0.05
	Borderline	474 (17.9)	308 (19.2)	166 (16)		
	Abnormal	209 (7.9)	134 (8.3)	75 (7.2)		
TG	Acceptable	1,595 (60.3)	916 (57)	679 (65.5)	19.53	0.001
	Borderline	677 (25.6)	442 (27.5)	235 (22.7)		
	Abnormal	371 (14)	249 (15.5)	122 (11.8)		

Data compared by chi-square test

## Discussion

In the present study, the measured mean TC was 152.5 mg/dl. The amount of measured TC for the 11-14 and 15-18 age groups were 154.9 and 148.8, respectively, with the highest value for girls (154.4 *vs.* 150). Based on a study on the adolescents of Tehran-Iran (during 2005-2008), the mean TC from 10-14 and 15-19 years old were determined at 162, 160, and 144, 154 mg/dl for boys and girls, respectively.^18^ The mean TC for the Americans aged 10-14 was reported to be 160 for both sexes.^19^ The same study on 10-18 year olds Korean adolescents indicated the mean TC of 159.^20^

According to our results, it can be pointed out that the average TC for both age groups of adolescents in Birjand is lower than the reported values in Tehran, America and Korea. Herein, the measured LDL for 11-14 and 15-18 years old of Birjand and their means were 84.8, 81.4, and 83.4 mg/dl, respectively. This quantity for 10-14 years old of Tehran was 95 mg/dl for both sexes, whilst for 15-18 year old boys and girls was 83 and 91, respectively.^18^ The measured LDL for American adolescents aged 10-14 years was 97 for both sexes.^19^ The mean LDL for 10-18 years old Korean adolescents was reported to be 89.^20^ The data clearly state that the mean LDL of Birjand adolescents is lower than those from Tehran, America and Korea.

The HDL value of 11-14 and 15-18 years old of Birjand adolescents and their mean HDL were measured at 48.1, 46, and 47.2 mg/dl, respectively. The mean HDL for boys and girls aged 10-14 and 15-18 years from Tehran were 46.8, 43.7, 39.8, and 44.2 mg/dl, respectively.^18^ According to available report, among the American adolescents, this quantity for 10-14 and 15-19 years old were 55, 52, 46, and 52 mg/dl for boys and girls, respectively.^19^ The HDL for the Korean 10-18 years old was measured at 52 mg/dl.^20^ The data clearly shows that the mean measured HDL of Birjand adolescents is higher than those from Tehran and lower than the American and Korean adolescents.

The TG values of the age groups 11-14 and 15-18 years old and their average were measured at 94.7, 88.1, and, 92.1 mg/dl, respectively. Similar quantity for the boys and girls from Tehran, aged 10-14 and 15-18 years old, and American’s were reported at 82, 95, 91, 84 and 63, 72, 78, 73 mg/dl, respectively.^18^^,^^19^ Based on a study on the Korean adolescents, the mean TG was reported at 89 mg/dl.^20^

It should be noted that the mean TG of Birjand is higher than the American and Korean adolescents are; while compared with those from Tehran, this value is higher for the age group of 11-14 years old but the same for the 15-18 years old. 

It is observed that in Birjand, the mean TC and LDL for boy adolescents are higher than girls are. Statistically, the measured values for other lipids did not show significant differences for both sexes. Based on two studies on adolescents in America and Tehran, the mean TC and LDL of the 15-18 year olds were measured at a higher value for girls than boys, while there were the same between boys and girls aged 11-14 years.^18^^,^^19^ However, the TC values of the Korean girls were reported to be higher in comparison with boys.^21^ Our results show that the mean TG and HDL of Birjand adolescents were higher and lower, respectively, in comparison with the American and Korean cases.

It is worth mentioning that our data on adolescents of Birjand is similar to many other studies in Iran.^16^ The results of a study on 4,824 students aged 6-18 from six Iranian cities (Tabriz, Rasht, Gorgan, Mashhad, Yazd, and Tehran)^16^ and a few other related studies showed that the mean TG and HDL of the Iranian adolescents were respectively higher and lower in comparison with the adolescents from America and many other countries.^16-24^

In a study by Kelishadi that investigated 2,000 students in Esfahan, it was found that the TC, TG, and mean LDL were higher; and HDL was lower than those in the USA were. She compared the results of two investigations in 1993 and 1999 and revealed an increase of TC, LDL, and HDL levels.^25^

Additional investigations by Kelishadi covering students aged 6-18 years old indicated that the means of TG, TC, LDL, and HDL of the Iranian children and adolescents were respectively higher and lower than the standard levels of Lipid Research Clinic (LRC).^26^ The findings of the present study are in agreement with the results by Kelishadi. Based on our study, 34.2% of adolescents had at least one dyslipidemia. The HDL of 42.6% of the individuals was lower than 45 mg/dl, in 39.6% of the cases TG≥100 mg/dl, in 24% of the cases TC≥200 mg/dl, and in 11.2% of the cases LDL≥100 mg/dl. Our results showed that, boys had lower HDL and higher TG than girls. The difference in other dyslipidemia between males and females would not have been significant. Concurrent with our results indicating low HDL being the most common dyslipidemia, high prevalence of low HDL among children and adolescents has been reported in the majority of other Iranian studies.^16^^,^^18^^,^^23^^-^^27^

Various prevalence rates of dyslipidemia in children have been reported in different studies. Genetic, racial, and environmental differences lead to a variation of lipid levels in children. Moreover, diverse definitions of dyslipidemia and the use of different limits for normal and abnormal ranges in the various studies should be taken into account. Consequently, comparison of the prevalence of dyslipidemia between different studies is a challenge. According to a study during 2005-2008 on adolescents of Tehran (aged 10-14 years old), it is noted that the prevalence of dyslipidemia; including high TC, low HDL, High LDL, and high TG were 11.8%, 35.5%, 10.1%, 5.7% for girls and 8.5%, 25.2%, 8.8%, 4% for boys, respectively. Similar values for girls and boys aged 15-19 years were 8.7%, 35.9%, 7.7%, 4.1% and 3.7%, 54.1%, 3.6%, 8.9%, respectively. In another study, almost half of the adolescents in Tehran, aged 10-19, had low HDL.^18^

Referring to the study by Kelishadi on students of 23 Iranian provinces aged 6-18 years, it is concluded that 45.7% of individuals had dyslipidemia and low HDL, hypertriglyceridemia, hypercholesterolemia, and high LDL were observed in 24.8%, 24.5%, 6.4%, and 6.3% of the cases, respectively.^26^ She stated that the prevalence of high TC and LDL are higher in the western countries since high TC and LDL in American adolescents are 26% and 20%, respectively.^28^ Similar values for adolescents in Brazil were 27.9% and 26.4%, respectively.^29^

The prevalence of the components of metabolic syndrome in children and adolescents of Europe, Asia, and South America were compared in a large-scale study. It is shown that the prevalence of dyslipidemia among the Iranian and Brazilian youth is significantly higher than the Germans. Particularly, the prevalence of low HDL among the Iranian individual was much higher in comparison with the Germans (38% *vs.* 7%).^30^

Yang performed a study in accordance with the criteria set by the *National Cholesterol Education Program* (NCEP) on the Korean children and adolescents aged 10-18 years old.^21^ It is indicated that 19.7% of the cases had at least one type of dyslipidemia. This result is extremely close to its American version (20.3%) but the prevalence of hypercholesterolemia was lower and the prevalence of low HDL was higher.^19^

Unlike in the USA, the prevalence of hypercholesterolemia and high LDL was more among the Korean females.^21^ Similar prevalence was observed in few other Asian countries such as Iran.^23^ Another study on the Korean students aged 10-18 years old, showed that the prevalence of dyslipidemia in girls and boys was 21.7% and 25.2%, respectively.^31^ A study conducted on individuals from two regions of Argentina, aged 7-14 years old, revealed the prevalence of high TG in 28.8% and 3.5% and low HDL in 30% and 5.5% of cases.^31^ Improving the level of the mean serum lipids of the American children has been reported by some studies; such as the National Health and Nutrition Examination Survey (NHANES) that was performed during 1988-1994 and 2007-2010.^32^ A study on the Korean adolescents revealed that the prevalence of low HDL has increased during 1998 to 2005 following a decrease from 2005 to 2008.^33^

In an investigation by Hosseini *et *al., three surveys (during 1999-2001, 2002-2005, and 2006-2008) on the children and adolescents of Tehran aged 10-19 years old were conducted and compared. The outcome of those surveys indicated that; (i) the total prevalence of high LDL decreased from 15% to 7.4%, (ii) the study on the age group 10-14, indicated an increase of the mean HDL and decrease of the low HDL prevalence, and finally (iii) during these periods, considerable increase in the overweight prevalence had occurred.^18^

The present study had some limitations. The blood test for lipid screening was only carried out once, although two blood tests were recommended. In addition, factors associated with dyslipidemia and dietary of adolescents were not mentioned. The advantages of the present research are; a study with a large population sample, representative sampling methodology, and data collection according to the standardized protocols. 

Regarding the findings of the present study, the means of TC, LDL, and HDL among the adolescents of Birjand were lower than the American reference values and the mean TG was higher than that. The reasons for the high prevalence level of dyslipidemia among the adolescents of Birjand could be due to the racial differences in serum lipid levels, high-fat diet, and sedentary lifestyle. An increase in the prevalence of obesity among the children of Birjand has also been reported.^34^ A planning for regional lipid percentiles of the Iranian children regarding the diagnosis of dyslipidemia is recommended. Lipid screening of adolescents, in particular those in high-risk conditions and an accurate follow up of individuals suffering from dyslipidemia is also advised.

Further studies to identify the influencing factors in the onset of dyslipidemia and the intervention measures to control the problem are essential. Correcting for the lifestyle, healthy nutrition, and encouraging adolescents to exercise, more mobility, and preventing obesity in adolescents are crucial. Pursuing appropriate state health policies is necessary to prevent the problem. Periodical studies in the years ahead to investigate dyslipidemia prevalence trend in children is also recommended. 

## Conclusion

According to the findings of the present study, dyslipidemia has a high prevalence in adolescents of Birjand. This could be due to the changes in diet, increased consumption of fast food instead of traditional food, sedentary lifestyle, and increasing obesity in adolescents. 
